# Pathogenicity of *NUSAP1* Variants Is Defined by NMD‐Escape: Evidence From Two Novel Cases and Systematic Population‐Based Variant Analysis

**DOI:** 10.1111/cge.70244

**Published:** 2026-09-07

**Authors:** Maureen Jacob, Susann Badmann, Stefania Bigoni, Maria Iascone, Ilaria Vivaldi, Katharina Mayerhanser, Robert Kopajtich, Holger Prokisch, Cristina Forest, Sebastian Eck, Michael Zech, Elisabeth Graf, Matias Wagner, Juliane Winkelmann, Theresa Brunet, Melanie Brugger

**Affiliations:** ^1^ Institute of Human Genetics, School of Medicine and Health, Technical University of Munich Munich Germany; ^2^ Medical Genetics Unit, Department of Mother and Child and Department of Medical Science Ferrara University Hospital and Ferrara University Ferrara Italy; ^3^ Laboratory of Medical Genetics, Papa Giovanni XXIII Hospital Bergamo Italy; ^4^ Institute of Neurogenomics, Helmholtz Zentrum München Munich Germany; ^5^ Department of Medical Sciences, Pediatric Section University Hospital of Ferrara Ferrara Italy; ^6^ Institute for Advanced Study, Technical University of Munich Garching Germany

**Keywords:** microcephaly, nonsense‐mediated decay, NUSAP1, protein‐truncating variants

## Abstract

Protein‐truncating variants in the 3′ region of a transcript, evading mRNA degradation and giving rise to aberrant truncated proteins, are an underrecognized cause in Mendelian diseases. Here, we report two individuals with heterozygous *de novo* nonsense variants in the penultimate and last exon of *NUSAP1*, both presenting with early‐onset refractory epilepsy, global developmental delay, congenital microcephaly, and a recognizable facial gestalt. RNA sequencing performed in one individual did not show a reduction in expression, compatible with escape of aberrant transcripts from nonsense mediated mRNA decay (NMD). We systematically analyzed gnomAD population data to delineate a critical region at the 3′ region of *NUSAP1*, where nonsense variants introduce a premature termination codon and escape NMD. Such variants are absent from healthy controls, while frameshift variants producing C‐terminal elongations appear tolerated. This position‐dependent model provides guidance for diagnostic variant interpretation.

## Graphical abstract

Heterozygous *de novo* nonsense variants in the penultimate and last exons of *NUSAP1* were identified in two unrelated individuals, predicted to escape NMD. In population data, nonsense variants were observed in exons 1–9 (of 11) in *NUSAP1* but were absent from its 3′‐terminal region.

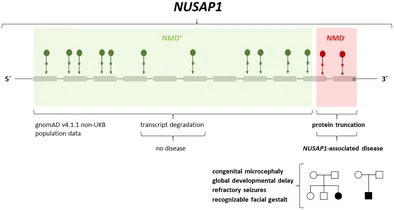

## 1 Introduction

Protein‐truncating variants (PTVs) located in the 3′ region of a transcript, specifically in the last exon or within the last 50‐55 nucleotides of the penultimate exon, may evade nonsense‐mediated mRNA decay (NMD), so‐called NMD‐escaping PTVs (PTVesc) [1, 2]. Additional PTVesc contexts include variants within the first 150 nucleotides of the first exon or within large exons exceeding 400 nucleotides [3, 4, 5, 6, 7]. We refer to these regions as the NMD^−^ region, while the remaining are considered NMD^+^, in which premature termination codons (PTC) are expected to trigger transcript degradation [8]. Rather than causing loss of function through transcript degradation, PTVesc variants may give rise to aberrant truncated proteins that exert gain of function or dominant negative effects or lead to other disruptions of protein interactions or localization [8].

A specific subgroup of such disease‐associated genes, in which escape of NMD is the underlying pathomechanism, is characterized by a high overall tolerance to PTVs, as reflected by low haploinsufficiency scores, while pathogenicity is restricted to PTVesc rather than PTVs as a class. These low scores lead to their systematic deprioritization during standard variant filtering pipelines such that PTVesc variants may be routinely overlooked, underscoring the need for refined variant interpretation strategies that explicitly incorporate variant position within the transcript and predicted NMD outcome [9]. It is currently estimated that hundreds of human genes may be susceptible to similar mechanisms, although most remain uncharacterized [8, 9].

*NUSAP1*, involved in the organization of the mitotic spindle, represents a prime example [10]. Mo et al. first described an association between heterozygous *de novo* variants in *NUSAP1* escaping NMD and a syndromic disorder comprising congenital microcephaly, seizures, brain abnormalities, and developmental delay in individuals carrying the identical nonsense variant located in the penultimate exon of *NUSAP1* [11].

In *NUSAP1*, all pathogenic variants identified to date lie within the NMD^−^ region, supporting the hypothesis that NMD escape is the relevant pathomechanism [9, 11]. By analyzing the distribution of PTVs at the 3′ end of *NUSAP1* observed in healthy individuals within gnomAD (gnomAD v4.1.1 non‐UKB), we were able to more precisely delineate the boundary between the NMD^+^ and NMD^−^ regions, providing a framework for variant interpretation in this gene [12].

Here, we report two additional patients with epilepsy, global developmental delay, microcephaly, and shared dysmorphic features harboring novel heterozygous *de novo* nonsense variants in *NUSAP1*, located in the penultimate and last exons, respectively.

## 2 Materials and Methods

Two unrelated individuals (ID#1 and ID#2) were recruited through the GeneMatcher platform [13]. DNA extracted from blood of patients and parents was used for trio exome (ID#1) or trio genome sequencing (ID#2); parental relationships were confirmed by trio analysis. RNA sequencing was done from skin fibroblasts of ID#1. Detailed information on sequencing methods and analysis of population data is provided in the Supporting Information Material and Methods.

## 3 Results

### 3.1 *NUSAP1* Variants

Trio exome sequencing in ID#1 and trio genome sequencing in ID#2 identified heterozygous nonsense variants in *NUSAP1*: NM_016359.5:c.1264C>T, NP_057443.2:p.(Arg422Ter) and NM_016359.5:c.1213C>T, NP_057443.2:p.(Gln405Ter), respectively (see Table 1 for variant details and Figure S1 for a regional tolerance landscape of *NUSAP1*). For both cases, the variant was not detected in parental blood DNA, suggesting a *de novo* origin. Detailed information regarding the prioritization and classification of *NUSAP1* variants can be found in the Supporting Information Results. According to current ACMG guidelines, both *NUSAP1* variants are classified as *likely pathogenic* (PS2, PM2_supporting, PM4) [14].

**TABLE 1:** Genetic and clinical information of affected individuals.

	#1	#2	II‐2	II‐1
	This study	This study	Mo et al	Mo et al
Sex	Female	Male	Male	Male
Age at last assessment	12 years 8 months	12 years 8 months	4 years 11 months	3 years 1 months
Age of death	n.a.	n.a.	n.a.	n.a.
cDNA (NM_016359.5)	c.1264C>T	c.1213C>T	c.1209C>A	c.1209C>A
Protein (NP_057443.2)	p.(Arg422Ter)	p.(Gln405Ter)	p.(Tyr403Ter)	p.(Tyr403Ter)
Genomic position GRCh38 (NC_000015.10)	g.41380124C>T	g.41377285C>T	chr15:41377281:C:A	g.41377281C>A
gnomAD frequency (v4.1.0)	0	0	0	0
CADD (v1.6) score	40	47	35	35
GERP++ score	3.74	5.55	−1.84	−1.84
Inheritance	*de novo*	*de novo*	*de novo*	*de novo*
Zygosity	Heterozygous	Heterozygous	Heterozygous	Heterozygous
Consanguinity	No	No	No	No
Family history	No	Paternal aunt: still birth and anencephaly	Negative	Negative
Gestational age at birth (weeks)	38 + 3	36	40 + 2	Full‐term
Birthweight	2700 g (10th percentile)	1810 g (< 3rd percentile)	3010 g	2819 g
Head circumference at birth	30 cm (below 1st percentile)	27 cm (below 1st percentile)	30.5 (*Z* score—3.31)	Unknown, 34 cm at 3 months of age (< 5th percentile)
Microcephaly	Yes	Yes	Yes	Yes
Developmental delay	Severe	Severe	Severe	Severe
Intellectual disability	Profound	Profound	n.a.	n.a.
Behavioral abnormalities	Autistic behavior, auto‐aggressive behavior	Difficult eye contact, global severe behavioral abnormalities	n.a.	n.a.
Muscular hypotonia	Yes	Yes	Yes	Truncal hypotonia
Epilepsy	Yes	Yes	Yes	Yes
Types of seizures	Focal seizures, secondary generalization	Palpebral eyelid myoclonia, spasms, tonic–clonic seizures	Generalized tonic–clonic seizures, asymmetric spasms, and myoclonic seizures	Frequent clusters of focal clonic and generalized tonic–clonic seizure
Seizure onset	First days of life	8 days of life, drug resistant	Neonatal seizures	First day of life
Seizure frequency	2–15 times a day, while she is awake	At the beginning several times in a day, now 2–3 times a day	n.a.	n.a.
Response to seizure medication	Poor	Poor	Poor	Poor
Brain MRI abnormalities	Mild hypoplasia of the corpus callosum	Agenesis of corpus callosum, absence of septum pellucidum, fornix and posterior commisura, dilated lateral ventricles, pachygyria/lissencephaly, delayed myelination, cerebellar hypoplasia, enlarged cisterna magna	Gyral simplification, a rudi‐mentary corpus callosum, olfactory bulb hypoplasia, nearly absent Midline commissures, and sub‐ependymal nodules in the lateral ventricles	e.g., Gyral simplification, partial agenesis of the corpus callosum, periventricular and deep white matter heterotopias, aplasia of the olfactory bulbs and tracts, irregular shape of putamen and globuls pallidus, poorly folded hippocamoi, interhemispheric cyst
Disorder of the visual system	Severe myopia	n.a.	Cerebral visual impairment	Cerebral visual impairment
Hearing impairment	No	Bilateral mixed hearing loss	No	Yes
Skeletal features	Scoliosis	Scoliosis	n.a.	n.a.
Endocrinological findings	Hashimoto's thyroiditis, central precocious puberty	Central precocious puberty	n.a.	n.a.
Dysmorphic features	Flattened occiput, wide interocular distance, deep‐set eyes, sunken nasal root, flat nasal bridge, wide mouth with protruding teeth and enlarged interdental spaces, an inverted lower mucosal lip and protruding ears	Flattened occiput, sloping forehead, posterior rotated ears, prominent supraorbital ridges, short philtrum, spaced teeth, thick lips, hypertelorism, deep‐set eyes, depressed nasal root, prominent ears	Small forehead, prominent nose, high palate, small chin	Hypertelorism, forehead abnormality, flat face, upslanting palpebral fissures, anteverted nares, abnormal palmar creases

Abbreviaion: n.a. = not available.

### 3.2 mRNA Sequencing in Individual #1

mRNA sequencing was performed in ID#1 with no evidence of aberrant splicing or reduced expression of *NUSAP1* compared with controls (Figures S2–S4, see Supporting Information Results for further information on results of RNA analysis).

### 3.3 Phenotype of Individuals

ID#1 and ID#2 were born to healthy non‐consanguineous parents (see pedigrees in Figure 1). For both individuals, congenital microcephaly was reported.

**FIGURE 1:**
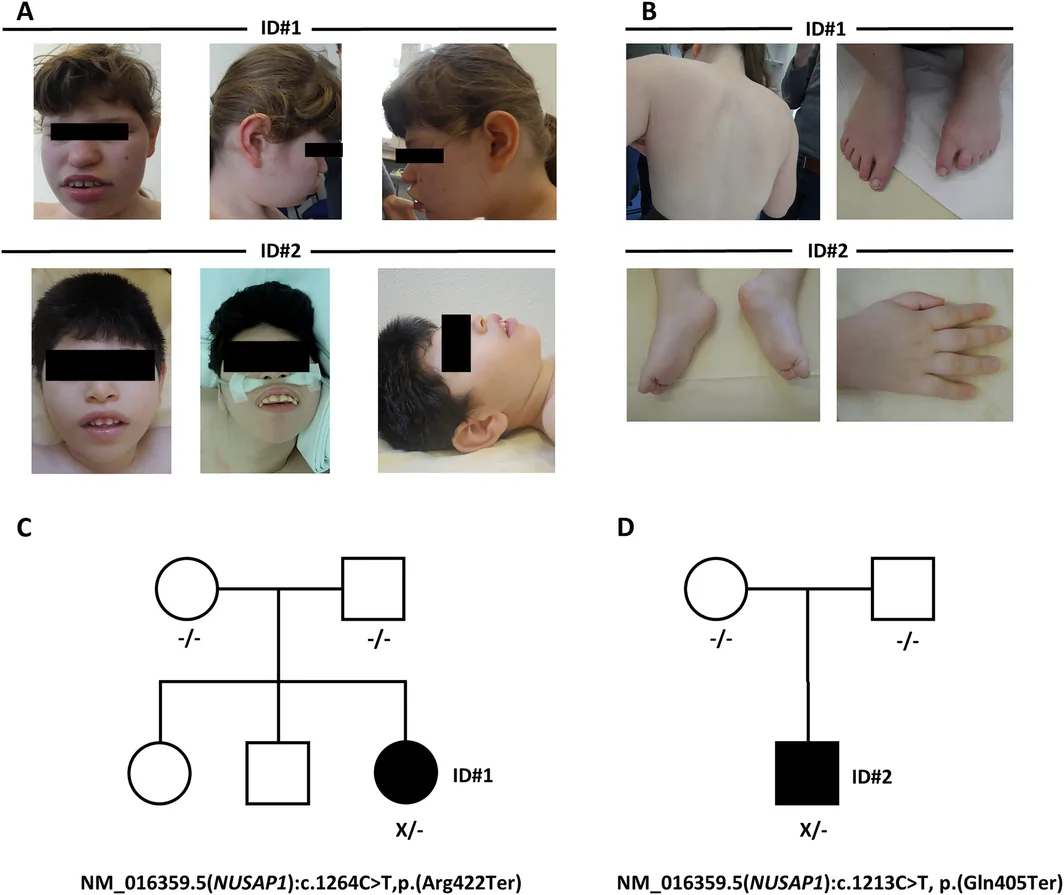
(A) Photographs of ID#1 and ID#2 both at the age of 12.8 years. (B) Photographs of the back of ID#1 showing scoliosis and feet showing an Egyptian foot. Photographs of ID#2 showing unremarkable feet and right hand showing broad and tapered fingers. (C, D) Pedigrees of family ID#1 (C) and ID#2 (D). Filled symbol = affected individual; circles = females; squares = males; −/− = homozygous wild‐type genotype, X/− = heterozygous genotype, with X representing the *NUSAP1* variant carried by the individual. [Colour figure can be viewed at wileyonlinelibrary.com]

Seizures began on the first day of life in ID#1 and on day 8 of life in ID#2, and remained refractory to treatment in both. Additional shared features were global developmental delay, profound intellectual disability, and variable abnormalities in brain MRI.

Clinical examination at age 12.8 years in both revealed microcephaly and a facial gestalt with hypertelorism, deep‐set eyes, depressed nasal root, flat nasal bridge, wide mouth with protruding widely spaced teeth, everted lower lip, and prominent ears.

Photographs of patients are provided in Figure 1. Clinical information is summarized in Table 1, showing a comparison to the previously reported cases [11]. The human phenotype ontology (HPO) was used to capture the patients' phenotype by reviewing clinical reports (Table S1) [15]. Detailed clinical case reports are provided in the Supporting Information Material.

### 3.4 Population Data

To further characterize the mutational landscape of *NUSAP1*, we analyzed PTV distribution across all exons in gnomAD v4.1.1 (non‐UKB) [12]. In exons 1–9 (of 11 exons), numerous PTVs are observed in healthy controls, including nonsense and frameshift variants, with no evidence of depletion.

This is in contrast to the variant distribution in the last exon and the 3′ end of the penultimate exon. Nonsense variants introducing a PTC within this region are absent in healthy controls. Frameshift variants are present, but uniformly generate C‐terminally elongated rather than truncated proteins. The frameshift variant NM_016359.5:c.1067del, NP_057443.2:p.(Pro356GlnfsTer22, termination codon at p.377), observed in gnomAD, establishes the minimal 3′ boundary of the NMD^+^ region in *NUSAP1*, while the variant NM_016359.5:c.1209C>A, NP_057443.2:p.(Tyr403Ter), reported in affected individuals, marks the minimal 5′ boundary of the NMD^−^ region [11]. The exact transition point between NMD^+^ and NMD^−^ within this interval of ~78 nucleotides cannot be determined.

The evaluated variants are listed in Table S2 and illustrated in Figure 2 (see Supporting Information Results for detailed information).

**FIGURE 2:**
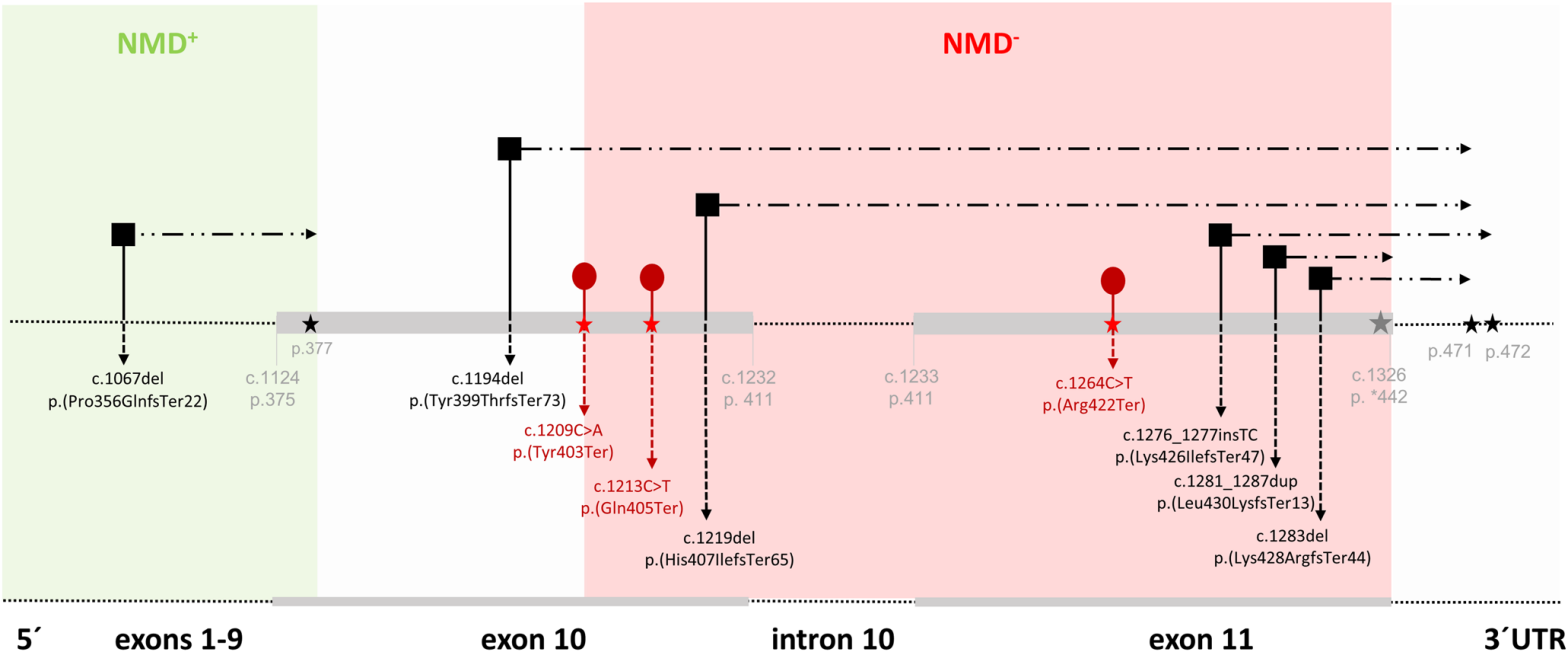
Schematic representation of the genomic organization of the penultimate and last exon of *NUSAP1* including variants reported in affected individuals and gnomAD (gnomAD v4.1.1 non‐UKB), from 5′ to the 3´end (NM_016359.5). Exons = grey boxes, introns = black dotted lines, size not to scale. Gray lines = exon‐intron boundaries. Frameshift variants identified in healthy controls = black box. Nonsense variants = red circles. Horizontal dashed arrow = length of the altered protein sequence. Black star = location of the stop codon generated by the variant. Grey star = canonical termination site. [Colour figure can be viewed at wileyonlinelibrary.com]

## 4 Discussion

Here, we report two additional individuals with novel heterozygous *de novo* nonsense variants in *NUSAP1*, expanding the mutational spectrum of this disorder. Consistent with the cases described by Mo et al. and the observations of Torene et al., our findings consolidate *NUSAP1* as a disease gene in which pathogenicity is restricted to PTVesc, and underscore the broader challenge of identifying such disease‐causing variants through standard variant interpretation pipelines [9, 11]. Our study provides photographs of affected individuals, revealing a consistent and recognizable facial gestalt across unrelated patients [11].

Identifying pathogenic variants in *NUSAP1* is particularly challenging given its pLI (probability of LoF intolerance) score of 0 and LOEUF (loss‐of‐function observed/expected upper bound fraction) of 0.81, both reflecting a high tolerance to loss‐of‐function variation and leading to systematic deprioritization of PTVesc in standard filtering pipelines [12, 16].

Systematic analysis of population data allowed us to define the minimal NMD^+^ and NMD^−^ regions within *NUSAP1*. All known pathogenic variants localize to the penultimate or final exon, consistent with NMD escape. Frameshift variants observed in healthy controls within these exons uniformly produce C‐terminally elongated proteins, suggesting that sequence alterations elongating the C‐terminal domain are tolerated. In contrast, nonsense variants generating PTC within this region are absent from controls and associated with disease.

RNA sequencing in ID#1 supports NMDesc of pathogenic *NUSAP1* variants by showing regular expression in fibroblasts compared with controls, although without protein‐based methods applied, the downstream effect of the variant has to be determined in further studies. The lack of additional patient‐derived fibroblast cell lines represents a limitation of our study, as confirmation of NMD escape in more cases has to be shown to generalize our findings. Since individuals affected by *NUSAP1*‐related disorder mainly present with a neurodevelopmental phenotype, investigating *NUSAP1* RNA and protein in neuronal cell types will be of high relevance.

The precise molecular mechanism by which PTVesc in *NUSAP1* cause disease remains to be elucidated. It is currently unclear why variants generating PTCs in the NMD^+^ region, which trigger transcript degradation, as well as frameshift variants producing elongated proteins, appear to be tolerated, while variants generating truncated proteins within the NMD^−^ region seem to be pathogenic. Mo et al. proposed that truncation of the *NUSAP1* C‐terminus removes the conserved charged helical domain and the mitotic centromere‐associated kinesin binding domain and may act through an antimorphic or gain‐of‐function mechanism. Functional studies addressing protein stability, protein interactions, and subcellular localization of the truncated *NUSAP1* protein will be essential to resolve this question.

Beyond variant position alone, our observations raise a broader question regarding the downstream consequences of frameshift variants within NMD^−^ regions. Frameshift variants in the 3′ region of *NUSAP1* observed in healthy controls do not introduce PTC within the NMD^−^ region, as the alternative reading frames of the final exon lack termination codons, resulting in C‐terminally elongated rather than truncated proteins. This suggests that PTVesc pathogenicity may depend not only on variant position relative to the NMD^+/−^ boundary, but also on termination codon distribution within the alternative reading frames, a parameter not yet systematically investigated in human disease. If generalizable, this principle could refine current models of PTVesc pathogenicity and inform future variant interpretation frameworks. Systematic investigation of alternative reading frame composition across NMD^−^ regions genome‐wide will be needed to evaluate this hypothesis.

## Author Contributions

M.J., T.B., and M.B. wrote the manuscript, analyzed the data, and interpreted the genetic findings. M.J., S.Ba., S.Bi., M.I., and I.V. performed clinical characterization of the patients. R.K., H.P., and M.Z. prepared and processed the RNA data. All authors reviewed and edited the manuscript.

## Funding

This study was funded by Else Kröner‐Fresenius‐Stiftung (2022_EKSE.185), Technical University of Munich–Institute for Advanced Study (to M.Z.), and Else Kröner excellence fellowship (2025_EKES.12) and Hertie Foundation (P1230037) (to M.W.).

## Ethics Statement

Sequencing for ID#1 was performed within a study approved by the local ethics committee in Munich (5360/121S). Sequencing for ID#2 was performed in a diagnostic context. All legal guardians of the patients gave their written consent for the genetic testing, clinical examination and publication of their data and agreed on the publication of (also facial) images. The study was conducted in accordance with the Declaration of Helsinki.

## Conflicts of Interest

The authors declare no conflicts of interest.

## Supporting information


**Table S1:** Listed are human phenotype ontology (HPO) terms used to capture ID#1's and ID#2's phenotype (1).
**Table S2:** Listed are frameshift variants located in the penultimate or last exon of *NUSAP1* that were observed in heterozygous state in unaffected individuals in the Genome Aggregation Database (gnomAD) v4.1.1 (non‐UKB) (2).
**Figure S1:** MetaDome tolerance landscape of *NUSAP1*.
**Figure S2:** Distribution of RNA‐seq expression values across samples.
**Figure S3:** *NUSAP1* variant in affected individual ID#1 and parents Visual illustration in the Integrative Genomics Viewer (IGV) of the variant detected in *NUSAP1* using trio whole‐exome sequencing.
**Figure S4:** Expression of *NUSAP1* in fibroblast RNA of affected individual ID#1 in comparison to four controls.

## Data Availability

The data that support the findings of this study are available on request from the corresponding author. The data are not publicly available due to privacy or ethical restrictions.
