# Supplementary material for: Pathogenicity of NUSAP1 Variants Is Defined by NMD‐Escape: Evidence From Two Novel Cases and Systematic Population‐Based Variant Analysis
Source: Clin Genet. 2026 Sep 7;110(6):768–74. doi: 10.1111/cge.70244 (PMC13728964; doi:10.1111/cge.70244)
Supplement: Supplementary file 1 [file CGE-110-768-s001.docx]

**Supplementary Table 1**

ID#1

| **HPO term** | **Symptom** |
| --- | --- |
| HP:0000154 | Wide mouth |
| HP:0000252 | Microcephaly |
| HP:0000316 | Hypertelorism |
| HP:0000545 | Myopia |
| HP:0000872 | Hashimoto thyroiditis |
| HP:0001250 | Seizures |
| HP:0001263 | Global developmental delay |
| HP:0002650 | Scoliosis |
| [HP:0002187](https://hpo.jax.org/browse/term/HP:0002187) | Intellectual disability, profound |
| [HP:0001252](https://hpo.jax.org/browse/term/HP:0001252) | Hypotonia |
| [HP:0000729](https://hpo.jax.org/browse/term/HP:0000729) | Autistic behavior |
| [HP:0002079](https://hpo.jax.org/browse/term/HP:0002079) | Hypoplasia of the corpus callosum |

ID#2

| **HPO term** | **Symptom** |
| --- | --- |
| [HP:0001511](https://hpo.jax.org/browse/term/HP:0001511) | Intrauterine growth retardation |
| [HP:0001562](https://hpo.jax.org/browse/term/HP:0001562) | Oligohydramnios |
| HP:0001263 | Global developmental delay |
| HP:0001250 | Seizures |
| [HP:0002187](https://hpo.jax.org/browse/term/HP:0002187) | Intellectual disability, profound |
| [HP:0001252](https://hpo.jax.org/browse/term/HP:0001252) | Hypotonia |
| [HP:0200136](https://hpo.jax.org/browse/term/HP:0200136) | Oral-pharyngeal dysphagia |
| [HP:0000410](https://hpo.jax.org/browse/term/HP:0000410) | Mixed hearing impairment |
| HP:0002650 | Scoliosis |
| [HP:0000826](https://hpo.jax.org/browse/term/HP:0000826) | Precocious puberty |
| [HP:0001274](https://hpo.jax.org/browse/term/HP:0001274) | Agenesis of corpus callosum |
| [HP:0000322](https://hpo.jax.org/browse/term/HP:0000322) | Short philtrum |
| [HP:0000687](https://hpo.jax.org/browse/term/HP:0000687) | Widely spaced teeth |
| HP:0000252 | Microcephaly |
| HP:0012433 | Abnormal social behavior |

**Caption Supplementary Table 1: Listed are human phenotype ontology (HPO) terms used to capture ID#1’s and ID#2’s phenotype (1).**

**Supplementary Table 2**

| **cDNA (NM_016359.5)** | **Protein change (NP_057443.2)** | **hg19** | **hg38** | **stop codon** | **Allele Count** | **Allele Frequency** |
| --- | --- | --- | --- | --- | --- | --- |
| c.1067del | p.(Pro356GlnfsTer22) | 15-41667968-AC-A | 15-41375770-AC-A | p.377 | 1 | 1.59e-6 |
| c.1194del | p.(Tyr399ThrfsTer73) | 15-41669463-TC-T | 15-41377265-TC-T | p.471 | 1 | 1.78e-6 |
| c.1219del | p.(His407IlefsTer65) | 15-41669485-AC-A | 15-41377287-AC-A | p.471 | 2 | 2.83e-6 |
| c.1276_1277insTC | p.(Lys426IlefsTer47) | 15-41672334-A-ATC | 15-41380136-A-ATC | p.472 | 1 | 1.66e-6 |
| c.1281_1287dup | p.(Leu430LysfsTer13) | 15-41672338-C-CAAAGGTT | 15-41380140-C-CAAAGGTT | p.442 | 1 | 1.66e-6 |
| c.1283del | p.(Lys428ArgfsTer44) | 15-41672338-CA-C | 15-41380140-CA-C | p.471 | 2 | 3.32e-6 |

**Caption Supplementary Table 2: Listed are frameshift variants located in the penultimate or last exon of *NUSAP1* that were observed in heterozygous state in unaffected individuals in the Genome Aggregation Database (gnomAD) v4.1.1 (non-UKB) (2). For each variant, the predicted protein-level consequence, the chromosomal position in hg19 and hg38, the resulting stop codon position, and the allele count and allele frequency in gnomAD are provided. Variants are listed in order of their nucleotide position within the transcript.**

**Supplementary Material and Methods**

**Cell culture of patient derived fibroblasts ID#1**

Patient-derived primary fibroblasts (ID#1) cell lines were maintained in DMEM medium-high glucose (D5786), supplemented with 10% fetal bovine serum (FBS), 100 U/ml penicillin and 100 µg/ml streptomycin. All cells were cultured at 37 °C supplemented with 5% CO2.

**Exome sequencing ID#1**

**The DNA library was prepared using the Twist Human Exome Kit 2.0, Comprehensive Exome Spike-In and Mitochondrial panel and s**equenced on an Illumina NovaSeq6000 system (Illumina, San Diego, California, United States) to an average of more than 72-fold coverage as 100 bp paired end run (>97% of the target sequences are sequenced at least 20 times). Alignment of the reads was performed to the University of California, Santa Cruz human reference assembly GRCh37 (hg19). SAMtools was used to detect single nucleotide variants, small deletions and insertions. Exome Depth was used for the detection of copy number variants as implemented in our certified (ISO 15189:2012) in-house pipeline EVAdb. Variants were analysed using EVAdb (https://github.com/mri-ihg/ngs_pipeline/tree/1.0.0).

**RNA sequencing**

RNA sequencing was conducted using RNA extracted from fibroblasts of ID#1 following the method outlined in a previous study (3). Fibroblast RNA was isolated using the RNeasy mini kit (Qiagen) following the manufacturer’s instructions. The RNA integrity number (RIN) was assessed with the Agilent 2100 BioAnalyzer (RNA 6000 Nano Kit, Agilent). Strand-specific RNA-seq was carried out as per the TruSeq Stranded mRNA Sample Prep LS Protocol (Illumina). Briefly, 1 μg of RNA was purified with poly-T oligo-attached magnetic beads and fragmented. The RNA fragments were reverse-transcribed using the First Strand Synthesis Act D mix, and second-strand cDNA was synthesized with Second Strand Marking Mix. The double-stranded cDNA was then subjected to end repair, A-tailing, adaptor ligation, and library enrichment. RNA libraries were sequenced as 100 bp paired-end reads on the Illumina HiSeq6000 platform. The RNA-seq reads were demultiplexed and mapped to the hg19 genome assembly using STAR v2.7.0a, applying default parameters and enabling the "Basic" mode for two-pass mapping to detect novel splice junctions. Aberrant gene expression was analyzed using the OUTRIDER pipeline with Z-scores calculated based on normalized counts (4). To detect aberrant expression and splicing, a control cohort of 197 strand-specific fibroblast RNA-samples from patients with Mendelian disorders, as previously described, was used (5, 6).

For ID#1, the estimated contamination level was 0.00723. The mean sequencing depth was 110.46, with 50.32% of target sequences covered at ≥20×. At the variant position in *NUSAP1* for ID#1, RNA-seq yielded a total read depth of 221 reads, of which 43% supported the variant allele (variant allele frequency [VAF], 0.43).

**Analysis of population data**

Frameshift and nonsense variants in *NUSAP1* were extracted from gnomAD v4.1.1 (non-UKB) and categorized by their position relative to the canonical termination codon and penultimate exon-exon junction, predicted protein impact, allele frequency, and observed zygosity (2). Additionally, the ClinVar database was searched for variants reported in *NUSAP1*, using filters “missense,” “nonsense,” and “frameshift”, irrespective of their pathogenicity.

**Supplementary Results**

**Variant interpretation in ID#1 and ID#2**

No additional variants were prioritized in both cases, as none of the other identified variants met criteria for *likely pathogenic* or *pathogenic* classification according to ACMG criteria or showed a convincing association with the patients’ phenotypes (7).

According to current ACMG guidelines, both *NUSAP1* variants are classified as likely pathogenic (PS2, PM2_supporting, PM4). PVS1 was not applied, since currently there is no evidence of loss of function as the underlying disease mechanism in *NUSAP1*-related disorder (8). Instead, PM4 was applied to reflect the assumed protein-truncating effect of the variants (9). PM2 was downgraded to PM2_supporting, in line with the current ClinGen SVI recommendation (10). In summary, *NUSAP1* variants were considered causative for the patient’s phenotype.

**Analysis of RNA sequencing data in ID#1**

Unbiased OUTRIDER analysis identified three significant expression outliers in genes not currently associated with disease (PPAN, PPAN-P2RY11, RNF26). *NUSAP1* was not a significant expression outlier and showed normal expression compared to control samples (Supplementary Figure 2) (4). The respective *NUSAP1* variant was detectable in 43% of reads (Supplementary Figure 3) compatible with escape of aberrant transcripts from nonsense-mediated mRNA decay (NMD). Analysis for splicing outliers showed no evidence of aberrant splicing of *NUSAP1* (Supplementary Figure 4) (6).

**Population data and ClinVar**

ClinVar lists no frameshift or nonsense variants classified as *benign* or *likely benign* across *NUSAP1*. Notably, the frameshift variants reported here that are observed in healthy individuals in gnomAD are not listed in ClinVar (11).

**Supplementary case reports**

**Case Report ID#1**

The patient is the third child of healthy, non-consanguineous parents. Pregnancy was complicated by placenta praevia with bleeding at 12 weeks; prenatal sonography revealed microcephaly and mild ventriculomegaly. She was born at 38+3 weeks (birth weight 2700g, 10th percentile; head circumference 30cm, <1st percentile; APGAR 9/10/10). TORCH screening and newborn screening were unremarkable.

Seizures began in the first days of life with apnea and hand twitching, followed by focal episodes with head retroflexion, rightward eye deviation, leg extension, and body stiffness, occurring up to 15 times daily. EEG showed isolated sharp waves over the right hemisphere. Despite treatment with phenobarbital and levetiracetam, seizures remained refractory. Brain MRI at three months revealed mild hypoplasia of the corpus callosum.

Early motor and language milestones were delayed, with unaided sitting at 11 months and babbling at 14 months, followed by developmental regression. By age 12, she exhibited autistic traits, auto-aggressive behavior, and incontinence. Additional findings include severe myopia, scoliosis requiring spinal fusion (TH5–L3) at age 11, Hashimoto's thyroiditis, and vitamin D deficiency. She attends a specialized educational institution and receives physiotherapy and occupational therapy.

At age 12.8 years (height 140cm, weight 40kg), clinical examination revealed microcephaly with flattened occiput, hypertelorism, deep-set eyes, depressed nasal root, flat nasal bridge, wide mouth with protruding teeth and enlarged interdental spaces, everted lower lip, and prominent ears.

Prior genetic workup including karyotype, FISH for 15q11.2q13, array CGH, and an epilepsy gene panel was unremarkable. A heterozygous *TREX1* variant (c.412G>A) of uncertain significance was identified but found to be inherited. Trio-ES identified no diagnosis among OMIM-listed disease genes; however, analysis of rare *de novo* variants revealed a heterozygous nonsense variant in NUSAP1 (NM_016359.5: c.1264C>T) in the final exon, absent from published literature.

**Case Report ID#2**

A 12-year-8-month-old male, born to non-consanguineous parents of Italian and Brazilian descent, presented with a complex clinical phenotype. The pregnancy was complicated by intrauterine growth restriction and oligohydramnios, and severe congenital microcephaly was noted at birth. Postnatally, he exhibited marked muscular hypotonia and oropharyngeal dysphagia. His global developmental delay is profound, with severe motor and speech impairments and a profound intellectual disability. He has difficulty with eye contact and severe behavioral abnormalities, including self-injurious behavior.Seizures began on the eighth day of life and remain drug-resistant, with myoclonic, spasm, and generalized tonic-clonic seizures occurring 2–3 times daily. He also has a movement disorder characterized by dyskinesia and afinalistic movements. Despite these challenges, he attends a mainstream school with the support of a special needs teacher.Additional clinical findings include bilateral mixed hearing loss, mild hepatomegaly, scoliosis, and precocious puberty.

The combination of congenital microcephaly, severe developmental delays, epilepsy, and multisystemic involvement strongly suggested a genetic etiology, warranting comprehensive genetic testing. An epilepsy-panel diagnostic revealed a “variant of uncertain significance” in the gene *ATP12A2* in a heterozygous state, which is maternally inherited. This was followed by a trio whole genome sequencing (WGS), which revealed a heterozygous nonsense variant in *NUSAP1* that was not detectable in the parent’s DNA (*de novo* origin).

**Supplementary Discussion**

For both cases, the underlying variants were suspected to be of *de novo* origin, although low-level parental mosaicism and germline mosaicism cannot be ruled out completely. Therefore, we provided an empirical recurrence risk of 1-2% to the families (12).

To minimize the risk of misclassifying disease-causing variants as benign when defining the minimal NMD^+^ and NMD^-^ regions within *NUSAP1*, as the full gnomAD datasets includes UK biobank samples also from affected inidivuals, we used gnomAD v4.1.1 non-UKB (2). Notably, the UKB-inclusive version contains four variants all causing PTC at p.388. This suggests the boundary of the NMD^+^ region could extend at least to p.388. Given the rule that PTCs located in the 50-55 nucleotides of the penultimate exon may be PTVesc, the boundary deliniating the NMD^-^ region would be at ~p.392. Precisely defining this boundary will require additional healthy control individuals.

A recent study by Raemaekers et al. detailed a *de novo* paracentric inversion inv(15)(q14q15) in a girl with cleft palate, ASD, hypotonia, and mild growth restriction, featuring breakpoints in intron 8 of *MEIS2* and intron 7 of *NUSAP1*, yielding three fusion transcripts. Transcriptome analysis showed normal *MEIS2* expression but *NUSAP1* expression was reduced to 0.61-fold. The phenotype aligned primarily with *MEIS2*-related CPCMR syndrome (cleft palate, cardiac defects, intellectual disability; OMIM#600987), lacking microcephaly or epilepsy, seen in truncated *NUSAP1* cases. Notably, she was born at 39+6 weeks with a head circumference of 35 cm (54th percentile) and by age 3 years showed head circumference at -2 SD (slower postnatal growth but not meeting microcephaly criteria < -2/-3 SD). This case report is in line with our hypothesis that *NUSAP1* haploinsufficiency does not cause microcephaly, as postnatal head growth remained subnormal rather than congenitally reduced (13).

**Supplementary Figures**

**Supplementary Figure 1: MetaDome tolerance landscape of *NUSAP1*.** The MetaDome web server was used to visualize regional tolerance to missense variation across the NUSAP1 protein (14). The N-terminus is displayed on the left and the C-terminus on the right. Amino-acid positions are color-coded according to their predicted tolerance to variation, ranging from variation-intolerant (red) through neutral (yellow) to variation-tolerant (blue). The positions of the three disease-associated *NUSAP1* variants are indicated at the C-terminal region of the protein (p.403, p.405 and p.422). Overall, the C-terminal region shows a higher degree of predicted intolerance to variation compared with the more 5´regions.

**Supplementary Figure 2: Distribution of RNA-seq expression values across samples.** The violin plot depicts normalized, log-transformed *NUSAP1* RNA-seq expression values across 197 samples (196 controls and ID#1). Each dot represents one sample. The width of the violin reflects the relative density of samples at a given expression level, and the central line indicates the median expression value. The RNA-seq sample from individual ID#1 is highlighted in red.

**Supplementary Figure 3: *NUSAP1* variant in affected individual ID#1 and parents** Visual illustration in the Integrative Genomics Viewer (IGV) of the variant detected in *NUSAP1* using trio whole-exome sequencing. In the top panel, the cytogenetic localization of *NUSAP1* (red line) is displayed on chromosome 15 (15q15.1). The bottom panel shows the reference sequence of *NUSAP1*. Grey bars represent next generation sequencing (NGS) reads aligned to the respective sequence. The first track displays the patient ID#1, followed by the mother and the father. The bottom track shows the reads of the RNA sequencing of patient ID#1. In red, the heterozygous nonsense variant in the last exon (NM_016359.5:c.1264C>T; present in~50% of reads in ID#1), both parents do not carry the variant. The bottom track shows that the variant is found in ~43% of mRNA-reads of patient ID#1.

**Supplementary Figure 4: Expression of *NUSAP1* in fibroblast RNA of affected individual ID#1 in comparison to four controls.** Sashimi plots of RNA sequencing of fibroblast cell lines of individual ID#1 and four in-house controls shows splicing of exons 8-11 (from left to right) in *NUSAP1*. RNA sequencing demonstrates no obvious abnormal splice events.

**Supplementary References**

1.	Kohler S, Gargano M, Matentzoglu N, Carmody LC, Lewis-Smith D, Vasilevsky NA, et al. The Human Phenotype Ontology in 2021. Nucleic Acids Res. 2021;49(D1):D1207-D17.

2.	Karczewski KJ, Francioli LC, Tiao G, Cummings BB, Alfoldi J, Wang Q, et al. The mutational constraint spectrum quantified from variation in 141,456 humans. Nature. 2020;581(7809):434-43.

3.	Yepez VA, Mertes C, Muller MF, Klaproth-Andrade D, Wachutka L, Fresard L, et al. Detection of aberrant gene expression events in RNA sequencing data. Nat Protoc. 2021;16(2):1276-96.

4.	Brechtmann F, Mertes C, Matuseviciute A, Yepez VA, Avsec Z, Herzog M, et al. OUTRIDER: A Statistical Method for Detecting Aberrantly Expressed Genes in RNA Sequencing Data. Am J Hum Genet. 2018;103(6):907-17.

5.	Yepez VA, Gusic M, Kopajtich R, Mertes C, Smith NH, Alston CL, et al. Clinical implementation of RNA sequencing for Mendelian disease diagnostics. Genome Med. 2022;14(1):38.

6.	Mertes C, Scheller IF, Yepez VA, Celik MH, Liang Y, Kremer LS, et al. Detection of aberrant splicing events in RNA-seq data using FRASER. Nat Commun. 2021;12(1):529.

7.	Richards S, Aziz N, Bale S, Bick D, Das S, Gastier-Foster J, et al. Standards and guidelines for the interpretation of sequence variants: a joint consensus recommendation of the American College of Medical Genetics and Genomics and the Association for Molecular Pathology. Genet Med. 2015;17(5):405-24.

8.	Mo A, Paz-Ebstein E, Yanovsky-Dagan S, Lai A, Mor-Shaked H, Gilboa T, et al. A recurrent de novo variant in NUSAP1 escapes nonsense-mediated decay and leads to microcephaly, epilepsy, and developmental delay. Clin Genet. 2023;104(1):73-80.

9.	Abou Tayoun AN, Pesaran T, DiStefano MT, Oza A, Rehm HL, Biesecker LG, et al. Recommendations for interpreting the loss of function PVS1 ACMG/AMP variant criterion. Hum Mutat. 2018;39(11):1517-24.

10.	Harrison SM, Biesecker LG, Rehm HL. Overview of Specifications to the ACMG/AMP Variant Interpretation Guidelines. Curr Protoc Hum Genet. 2019;103(1):e93.

11.	Landrum MJ, Lee JM, Riley GR, Jang W, Rubinstein WS, Church DM, et al. ClinVar: public archive of relationships among sequence variation and human phenotype. Nucleic Acids Res. 2014;42(Database issue):D980-5.

12.	Kay AC, Wells J, Hallowell N, Goriely A. Providing recurrence risk counselling for parents after diagnosis of a serious genetic condition caused by an apparently de novo mutation in their child: a qualitative investigation of the PREGCARE strategy with UK clinical genetics practitioners. J Med Genet. 2023;60(9):925-31.

13.	Ek M, Kvarnung M, Pettersson M, Soller MJ, Anderlid BM, Thonberg H, et al. Multi-omics analysis detail a submicroscopic inv(15)(q14q15) generating fusion transcripts and MEIS2 and NUSAP1 haploinsufficiency. Sci Rep. 2024;14(1):30343.

14.	Wiel L, Baakman C, Gilissen D, Veltman JA, Vriend G, Gilissen C. MetaDome: Pathogenicity analysis of genetic variants through aggregation of homologous human protein domains. Hum Mutat. 2019;40(8):1030-8.
